# Effect of Bio-Based Flame Retardants in Sustainable Epoxy Systems for the Development of Composite Materials

**DOI:** 10.3390/polym17152001

**Published:** 2025-07-22

**Authors:** Patricia Ares-Elejoste, Rubén Seoane-Rivero, Inaki Gandarias, Jesus Ballestero, Ane Libe Alonso-Amondarain

**Affiliations:** 1 GAIKER Technology Centre, Basque Research and Technology Alliance (BRTA), Parque Tecnológico de Bizkaia, Edificio 202, 48170 Zamudio, Spain; ballestero@gaiker.es (J.B.); alonsoa@gaiker.es (A.L.A.-A.); 2 Chemical and Environmental Engineering Department, University of the Basque Country (UPV/EHU), Alameda Urquijo s/n, 48013 Bilbao, Spain; ruben.seoane@ehu.eus (R.S.-R.); inaki.gandarias@ehu.eus (I.G.)

**Keywords:** bio-based epoxy, bio-based flame retardants, sustainability, composites, transport sector

## Abstract

The composite materials industry is increasingly seeking sustainable alternatives to mitigate the environmental impact of end-of-life materials. As a result, many sectors are transitioning toward bio-based or partially bio-based matrices (e.g., epoxy resins) to preserve material properties while improving sustainability. The transportation sector, in particular, demands materials that meet stringent mechanical and fire resistance standards. In this study, various epoxy systems with bio-based and/or recyclable content were investigated, along with renewable additives designed to enhance fire resistance through their functional groups and chemical structure. The research focused on developing formulations compatible with Sheet Moulding Compound (SMC) technology, which is widely used in transportation applications. Through extensive testing, materials with high bio-based content were successfully developed, exhibiting competitive mechanical properties and compliance with key fire safety requirements of the railway sector, as per the EN 45545-2 standard.

## 1. Introduction

Climate change is one of the most pressing concerns of modern society, driving an increasing demand for sustainable alternatives in both products and production processes. Within the materials industry, particularly in the field of composites, considerable efforts are being made to develop environmentally friendly substitutes for traditional thermoset matrices. These composites have already contributed to reducing emissions by replacing heavier metallic materials in sectors such as transportation for several years [1,2]. However, despite their many advantages, thermoset composites pose a significant environmental impact at the end of their lifecycle, as they are primarily derived from fossil-based sources and are not recyclable. In response, companies specializing in thermosetting resin synthesis are increasingly turning to renewable raw materials to enhance the bio-based content of their products [3]. While these resin systems are not yet fully bio-based, they contribute to a significant reduction in greenhouse gas emissions compared to their fossil-derived counterparts. Among thermosetting resins, bio-based epoxy systems have garnered special attention due to their widespread industrial application and good mechanical properties [4]. The molecular structure of these resins allows the substitution of specific synthesis components with biomass-derived alternatives [4].

Beyond resin sustainability, industries have also been adopting eco-friendly reinforcements for several years. These reinforcements can be classified based on their origin: mineral (e.g., basalt), animal (e.g., lamb’s wool, cashmere), or plant-based (e.g., linen, jute, bamboo) [5]. In this study, basalt fibre has been selected due to its excellent mechanical and thermal properties, as well as its high chemical stability. Additionally, basalt fibre significantly reduces CO2 emissions and serves as a competitive alternative to glass fibre [6,7,8]. The manufacturing process employed in this research is the hand lay-up technique. However, following the completion of this study, the resulting formulations are expected to be adapted for processing using Sheet Moulding (SMC) technology, which is widely used in the transport sector for large-scale composite production.

Epoxy resins can be synthesized through two primary methods: (i) direct reaction between epichlorohydrin and phenolic compounds or bio-based carboxylic acid, and (ii) epoxidation of carbon–carbon (C-C) double bonds to form oxirane rings [2].

Several biomass-derived compounds, including cardanol, vanillin, lignin, and eugenol, have been identified as viable precursors for bio-based epoxy resins. However, it should be noted that these types of resins typically do not exceed 40% bio-based content, as higher concentrations may negatively affect properties such as glass transition temperature (Tg). In some cases, industries have also developed bio-based amine hardeners, further enhancing the sustainability of the overall system.

Although polymer-based composites offer notable advantages in terms of lightweight properties and mechanical strength, their high hydrocarbon content makes them highly flammable. The combustion process of these materials typically follows five stages: (i) sample heating, (ii) decomposition and emission of non-flammable and flammable gases, (iii) ignition of combustible gases, (iv) fire development, and (v) extinction [9]. To mitigate fire hazards, flame-retardant additives are incorporated into composite materials. These additives are categorized into two main groups: halogenated and non-halogenated compounds.

Halogenated flame retardants, particularly bromine-based compounds, are highly effective but have been increasingly restricted by environmental regulations such as REACH (Regulation on the Registration, Evaluation, Authorization, and Restriction of Chemicals), WEEE (waste electrical and electronic equipment) and RoHS (restriction of hazardous substances) due to their adverse effects on both the environment and human health [9,10,11]. Consequently, they have been replaced by nitrogen-, phosphorus-, and inorganic-based flame retardants such as trihydrated alumina (ATH). However, these halogen-free flame retardants often need to be added in larger quantities to meet the stringent fire safety requirements of sectors like transportation and construction [12].

Most commercially available flame retardants are petroleum-derived organic compounds, including non-halogenated phosphorus-based additives. The depletion of fossil resources and the growing emphasis on green chemistry have spurred interest in bio-based flame retardants as sustainable alternatives [13,14]. This study explores the use of bio-based flame retardants such as lignin, tannic acid, gallic acid, and phytic acid as additives for composite material manufacturing for the transport sector. The fire performance of these materials has been assessed by cone calorimetry, with additional evaluation against the railway sector standard EN 45545-2:2013+A1:2015: “Railway applications-Fire protection on railway vehicles-Part 2”. Brussels, 2020.

Although the EN 45545-2 standard mandates additional fire tests for certification, cone calorimetry serves as an effective preliminary screening tool. Among the fire performance parameters obtained from this test, the Maximum Average Rate of Heat Emission (MARHE) is especially critical for material classification in the railway sector. Additionally, the peak heat release rate (Qmax) provides valuable insight into fire behavior, aiding in the assessment of material safety. According to EN 45545-2, materials are classified based on their Requirement Set (R) and Hazard Level (HL) (see Table 1), with R1 and HL3 representing the most stringent criteria.

In the present study, bio-based formulations and composites were developed, employing epoxy systems with bio-based content alongside selected bio-additives to enhance their fire performance. Specifically, three bio-based epoxy resins, detailed in Section 2, were investigated. Following an evaluation of the reactivity and stability of these formulations, considering the targeted end-use application and the anticipated future manufacturing process, screening was conducted to identify the most suitable system. Subsequently, various bio-based additives were incorporated into the selected system, and it was confirmed that these additives did not significantly interfere with the curing reaction.

The bio-additives assessed included lignin and tannic acid (due to their high hydroxyl content—OH), phytic acid (due to its phosphorus content—P), and chitosan (due to its nitrogen content—N).

### 1.1. Lignin

Lignin is a natural substance that constitutes part of the cell wall in many plants, providing rigidity and resistance. While traditionally used for energy production via combustion, lignin has gained considerable interest as a bio-based additive for producing various materials, including coatings, reinforcing agents, and biofuels [16,17]. Its potential as a flame retardant is currently under investigation, primarily due to the high hydroxyl (-OH) content in its structure.

Lignin exhibits excellent thermal stability and good mechanical properties, which are attributed to its non-crystalline network structure. Despite its complex structure, lignin can undergo chemical modifications such as phosphorylation or nitrogenation to enhance its fire-retardant properties [18]. During combustion, lignin forms a char layer that protects the underlying material, while compounds like phosphorus can act as oxygen scavengers [19]. Lignin can be classified into four main types based on the pulping technology used [20,21,22,23]: kraft lignin, lignosulfonate, soda, and organosolv lignin. Specifically, this work focuses on kraft lignin to evaluate its fire-retardant properties; a detailed description follows.

Kraft Lignin: The kraft process is widely used in the paper industry. It involves the use of sodium hydroxide and hydrosulfide anions at temperatures around 150–170 °C. The resulting lignin may contain impurities such as sulfur compounds and sugars, leading to lower purity. However, lignin can be easily modified to improve its quality depending on the intended application.

### 1.2. Tannic Acid

Tannic acid is a non-toxic, inexpensive, and abundant polyphenolic compound characterised by hydroxyl groups attached to a benzene ring. In addition, it may contain other groups such as sugars and organic acids. This compound can be obtained from the bark and other parts of various tree species and seeds [14,24].

The aromatic structure of tannic acid confers chemical and thermal stability, as well as low thermal conductivity [25]. Although commonly used as an antioxidant, its ability to release heat during thermal degradation and form char during combustion has led to its investigation as a flame-retardant additive in recent years [26].

### 1.3. Phytic Acid

Phytic acid is a naturally occurring, biodegradable acid compound containing a high phosphorus content. It is derived from plants and fruits [14,27].

Containing approximately 28% phosphorus, phytic acid is a recyclable natural resource. Its phosphorus content, coupled with other properties, is driving its adoption as a flame-retardant additive [28,29]. During combustion, the release of phosphorus from phytic acid accelerates the carbonization of polymer matrix, leading to the formation of a carbonaceous layer that inhibits the emission of combustible gases.

### 1.4. Chitosan

Chitosan is a renewable, environmentally friendly, and non-toxic aminopolysaccharide obtained by deacetylating chitin, which is primarily found in crustacean shells. Its structure contains a large number of hydroxyl and amino groups [14,30,31].

Beyond its application in agriculture as a fungicide and in the wine industry to prevent wine spoilage, chitosan is being explored for enhancing fire performance due to its high nitrogen content. The numerous hydroxyl and amino functional groups present in its structure make it a promising candidate for development and research in flame retardancy, as the nitrogen contributes to reducing oxygen levels during combustion.

## 2. Materials and Methods

Throughout this section, the materials and methods used in this research are detailed.

### 2.1. Materials

#### 2.1.1. Bio-Based and/or Recyclable Epoxy Systems

Among the epoxy resins that have bio-based content and/or are recyclable, four commercial systems were studied: SR FireGreen 37 and SR GreenPoxy 33, supplied by SICOMIN Epoxy Systems (France, Pluguffan), and the Polar Bear^®^ resin system supplied by R*Concept (Barcelona, Spain). These were used as supplied and according to the manufacturer’s recommendations without any modification.

The most relevant physicochemical properties of these resin systems are shown in Table S1 of the Supplementary Information.

Regarding the hardeners, the ones recommended by the suppliers were utilized. In Table S2 (Supplementary Information), all the hardeners’ properties are shown.

#### 2.1.2. Bio-Based Additives

Lignin, tannic acid, phytic acid, and chitosan were studied and, according to the literature shown in the introduction section, could have good flame-retardant properties. The characteristics of these additives are summarized in Table S3 (Supplementary Information). All the additives used were supplied by Sigma-Aldrich (St. Louis, MO, USA).

Additionally, a conventional (non-biobased but environmentally friendly) flame retardant was used in order to compare its performance with the above-mentioned bio-based additives. This flame retardant is an ammonium polyphosphate (APP) and was supplied by Budenheim (Budenheim, Germany) with the commercial reference FR CROS484. Its main properties are summarized in Table S4 (Supplementary Information).

#### 2.1.3. Basalt Fibre

The reinforcement used was a bi-directional basalt fibre supplied by the company Kamenny Vek-Basfibre (Dubna, Russia) with the commercial reference TBR-600^®^ (Table S5, Supplementary Information).

### 2.2. Methods

#### 2.2.1. Preparation and Characterisation of Epoxy Formulations

Prior to the fabrication of the composites, the resin systems mentioned in Section 2.1 were prepared and characterised. Once characterised, screening was carried out, concluding in the use of SR FireGreen 37 as the resin to be flame retarded with the bio-additives, which, according to the technical data sheet (TDS), is already certified for use in components that require fire resistance. This was carried out to assess whether the incorporation of these additives would interfere with the curing reaction of the resin system.

In Table 2, the developed formulations of the epoxy systems are detailed.

Table 3 shows the formulations in which the bio-based flame retardants were incorporated. Specifically, 15 parts per hundred resin (phr) of bio-additives were added to observe their effect in the curing reaction more clearly.

##### Differential Scanning Calorimetry—Characterisation of the Formulations

The curing behavior and thermal properties of the epoxy formulations (Table 2) were characterized by Differential Scanning Calorimetry (DSC). The analysis determined the reaction enthalpy (ΔH), peak exothermic temperature, glass transition temperature (Tg), and degree of conversion (α). Measurements were performed using a METTLER Toledo DSC1 STARe system (Greifensee, Switzerland), with samples precisely weighed (accuracy ±0.0001 g) on a METTLER TG50 analytical balance. The experiments employed 40 µL sealed aluminum crucibles under a nitrogen purge (50 mL/min). A dynamic temperature program from −50 °C to 280 °C was applied to monitor the curing process.

##### Thermogravimetry (TGA)

Thermogravimetric analysis (TGA) was performed on the SR FireGreen 37 epoxy system and on the bio-based flame retardants. By means of this study, the degradation temperature of the different samples and the mass loss of them with temperature were obtained. In this way, information such as the percentage of moisture in the samples and other substances, e.g., the flame-retardant content in a resin, can be obtained. For this purpose, a TGA instrument TGA DSC1 from METTLER Toledo was used. The process conditions used were a temperature range from 25 °C to 600 °C or 800 °C, depending on the sample, and air as the atmosphere.

##### Rheology

Viscosity measurements were conducted using an Anton Paar MCR501 rheometer (Graz, Austria) equipped with a 25mm disposable parallel plate geometry at 25 ± 2 °C. A shear rate range of 100–300 s^−1^; was employed for standard measurements, while formulations containing chitosan or linseed oil—which exhibited lump formation and measurement difficulties—required a reduced shear rate range of 1–100 s^−1^ to obtain reliable data.

#### 2.2.2. Preparation and of Cured Epoxy Formulations

**Figure 1:**
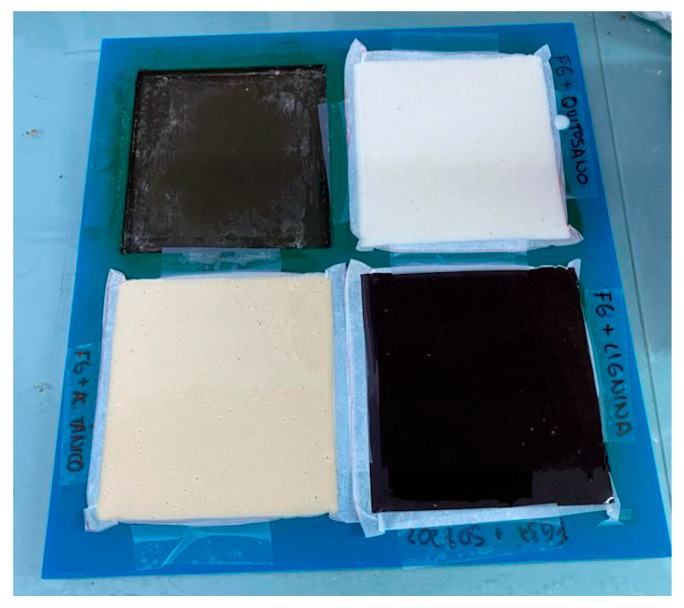
Silicon mould with samples made by casting.

The formulations listed in Table 3 were cast into 100 mm × 100 mm plates (4 mm thickness) using a four-cavity silicone mold (Figure 1). Silicone release paper was applied to the mold surfaces to facilitate demolding. Following the manufacturers’ technical specifications, the samples were initially cured at room temperature for 24 h, followed by a post-curing cycle of 24 h at 50 °C to achieve complete cross-linking.

To determine the fire properties of the bio-additives as flame retardants, an FTT cone calorimeter equipped with an O_2_/CO_2_/CO Ultramat/Oxymat 6 analyser from Siemens (Munich, Germany) with the capacity to simultaneously quantify the gaseous products was used. The purpose of this equipment was to quantify the amount of heat released by the sample when exposed to controlled levels of radiation. This test is governed by the standard ISO 5660-1:2015+AMD:2019: “Reaction to fire tests. Heat release, smoke production and mass loss rate”. Amendment 1. International Standard, Switzerland, 2019.

Specifically, the equipment can operate with radiation fluxes of up to 75 kW/m^2^, although, in this case, a radiation of 50 kW/m^2^ was used. By means of this test, the following results were obtained: Heat Release Rate (HRR), which was obtained by measuring oxygen consumption in the exhaust duct every 2 s; Average Rate of Heat Emission (ARHE); and Maximum Average Rate Heat Emission (MARHE), which is used as a parameter to classify the materials in the railway sector.

To carry out the characterisation, 100 mm × 100 mm samples were taken at a distance of 25 mm from the heated cone (60 mm in the case of intumescence). Before the sample was placed under the cone, it was wrapped in aluminum foil in order to avoid the possible detachment of residues during the test. Once wrapped, the sample was placed on a sample holder and placed under the cone on a load cell, whose function was to record the loss of mass of the sample during combustion.

The cone was then allowed to stabilise at a temperature of about 800 °C, and the heat shield was removed from the heat source so that the surface of the sample was irradiated while a lighter ignited the combustible gases released by the test specimen. Subsequently, as soon as the specimen ignited, the igniter was switched off and removed, at which time the ignition time was taken. Finally, the heat release rate was calculated by means of the oxygen consumption, which was initially at 20.95%. This O_2_ consumption was associated with an energy of 13.1·103 kJ/kg O_2_ consumed.

#### 2.2.3. Development and Characterization of Sustainable Composites

Composites were manufactured using hand lay-up to develop materials based on optimized formulations. In this technique, resin is applied to a mold (in this case, a flat wooden support coated with Teflon to facilitate demoulding), and layers of fibre are impregnated until the target thickness and grammage, as previously defined, are achieved.

The resulting sustainable composites were characterised by Differential Scanning Calorimetry (DSC) and cone calorimeter tests, following the procedures outlined in Section 2.2.1.

Additionally, in order to evaluate the mechanical properties of these composites, flexural tests were carried out. These tests were performed in accordance with the standard “UNE EN ISO 14125:1998/AC: Fibre-reinforced plastics composites. Determination of flexural properties”. Madrid, 2002. and were carried out in a universal testing machine made by Shimadzu (Kyoto, Japan), i.e., model AGX 100.

## 3. Results

The results obtained in the several tests carried out are discussed below.

### 3.1. Characterisation of the Resin Formulations

#### 3.1.1. Differential Scanning Calorimetry (DSC)

**Figure 2:**
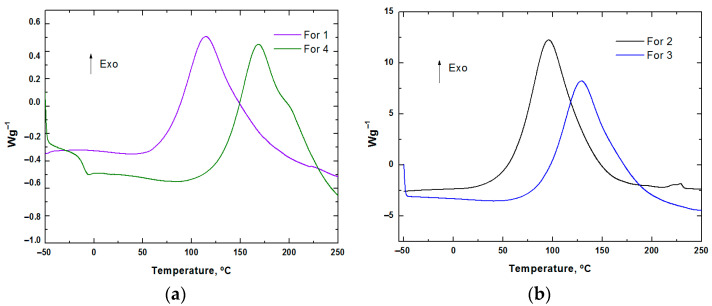
DSC graphs of (**a**) bio-based and/or recyclable and (**b**) bio-based formulations.

The formulations detailed in Section 2.2.1 (Table 2) were characterized by DSC, and the resulting graphs are presented in Figure 2.

In addition to the initial thermal analysis, reference samples were stored at room temperature to assess their stability over time (Table 4). This is particularly relevant, as these formulations are intended for processing via Sheet Moulding Compound (SMC) technology in future research to manufacture components for the transport sector, as mentioned in the Introduction.

Analysis of the data presented in Table 4 shows that all the formulations exhibit significant advancement in the curing reaction, with conversion rates exceeding 50% within 24–48 h of storage at room temperature. This indicates that under ambient conditions, these systems lack the stability required for effective SMC processing, where resin systems must remain processable over extended periods before compression moulding and curing.

From an industrial applicability perspective, these findings highlight the need to adapt the storage and handling protocols to ensure the formulations remain stable prior to moulding, especially under the stringent processing timelines and logistical constraints typical in the transport sector. Although Formulation 4 (Polar Bear system, bio-based and recyclable) exhibited comparatively higher stability after 48 h, it still showed partial conversion, indicating that even the most stable formulation requires cold storage for practical SMC application.

Notably, despite its lower stability, Formulation 1 (SR FireGreen 37 system) remains of particular interest due to its certification for fire-resistant applications, aligning with the safety and regulatory demands of the transport sector. In contrast, the SR GreenPoxy 33 system (Formulations 2 and 3) demonstrated both low stability and a lack of inherent fire-retardant properties, reducing its suitability for this application without additional modification.

For these reasons, Formulation 1 (containing SR FireGreen 37 resin) was selected for further development and optimization in subsequent tests, ensuring that its fire performance can be leveraged in the target application. To mitigate premature crosslinking and maintain processability in the SMC process, it will be essential to store the optimized formulation at sub-zero temperatures (approximately −18 °C) prior to moulding. Additionally, the glass transition temperature (Tg), a critical parameter for dimensional stability and thermal performance in transport components, was determined by DSC during a second dynamic scan for this system, providing baseline data for future processing and mechanical testing. The resulting Tg value is detailed in Table 5.

#### 3.1.2. Thermogravimetry (TGA)

By means of thermogravimetry tests, the degradation temperatures of each of the flame retardants were obtained. Additionally, TGA was conducted on the SR FireGreen 37 resin to identify potential additives responsible for its enhanced fire-retardant properties. As regards sample conditioning prior to analysis, samples were conditioned under laboratory conditions.

The following operating conditions were used to carry out this test:

- Temperature range: 25 °C to 600 °C or 800 °C, depending on the sample
- Atmosphere: Air
- Heating rate: 20 °C/min

**Figure 3:**
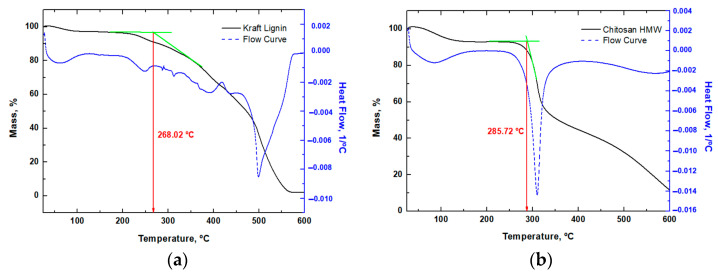

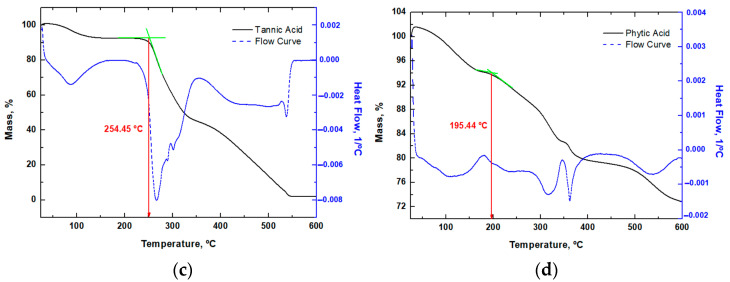
Thermal analysis results of (**a**) kraft lignin, (**b**) chitosan HMW, (**c**) tannic acid, and (**d**) phytic acid. The tangents in green indicated in the graphs have been used to calculate the onset.

The curve for kraft lignin (Figure 3a) indicates the onset of decomposition at approximately 268.02 °C. High molecular weight chitosan (derived from crustacean shells) exhibits an initial degradation temperature of approximately 285.7 °C (Figure 3b), suggesting slightly higher thermal stability compared to the analysed lignin.

With regard to tannic acid, the first step reflects an initial mass loss attributable to moisture (Figure 3c), with subsequent degradation occurring around 250 °C, a similar behaviour to that of chitosan. Phytic acid exhibits a lower degradation temperature (195 °C) compared to the other materials.

**Figure 4:**
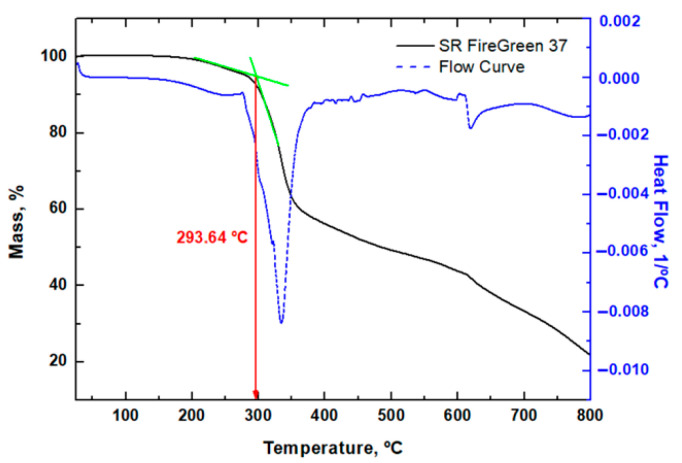
TGA results for SR FireGreen 37. The tangents in green indicated in the graphs have been used to calculate the onset.

On the other hand, the flame-retarded resin SR FireGreen 37 (Figure 4) reveals two distinct stages of decomposition at elevated temperatures. The initial mass loss occurs around 325 °C, suggesting the presence of ammonium polyphosphate (APP) as a flame-retardant additive, as APP typically decomposes between 275–300 °C [32]. The second step corresponds to the complete degradation of the resin.

#### 3.1.3. Rheology—Determination of the Viscosity of the Formulations

Prior to the composite development, the viscosities of the flame-retarded formulations detailed in Section 2.2.1 (Table 3) were determined, obtaining the following results:

As shown in Table 6, the viscosity of the chitosan-containing formulation (For 1.2) increased significantly, despite adjusting the shear rate. This is attributed to the presence of agglomerates in the sample, rendering the obtained viscosity value unreliable.

The remaining formulations exhibited higher viscosities than the reference (890–900 mPa∙s) but remained within a suitable range for fibre impregnation, approximately 1500 mPa∙s (without additives) to 10,000 mPa∙s (with additives), and potentially up to 50,000 mPa∙s.

#### 3.1.4. Cone Calorimeter—Fire Performance of the Cured Formulations

Among the coupons produced via casting, Formulation 4 (incorporating the Polar Bear system) was tested in the cone calorimeter to evaluate its performance, despite the absence of flame-retardant additives. This decision was justified by its superior stability, as discussed in Section 3.1.1.

The results detailed in Table 7 indicate that the fire performance of For 4 is not suitable for railway applications. This is evident from the MARHE value, which exceeds the railway sector requirement of 90 kW/m^2^. Furthermore, the sample exhibited dripping during combustion, and no intumescence was observed. This is related to the absence of the fire-retardant additives in the tested system, leading to significantly reduced fire resistance.

For the SR FireGreen 37 system and the flame-retarded formulations, the following results were obtained:

As can be seen in the table below (Table 8), all the formulations, including the reference (For 1), meet the MARHE requirement of <90 kW/m^2^. The addition of ammonium polyphosphate (FR CROS484) (For 1.1) and lignin (For 1.3) slightly increased the MARHE value, indicating a potential reduction in fire performance. Nevertheless, lignin was retained in subsequent formulations due to its bio-based origin and increasing market presence.

In contrast, the incorporation of chitosan, tannic acid, and phytic acid resulted in a slight improvement in the MARHE value compared to For 1. This is attributed to the functional groups present in these additives. Chitosan contains -N groups, which reduce oxygen levels and inhibit combustion. Tannic acid (-OH groups) and phytic acid (-P groups) work synergistically to dehydrate the matrix and promote the formation of a protective char layer.

**Figure 5:**
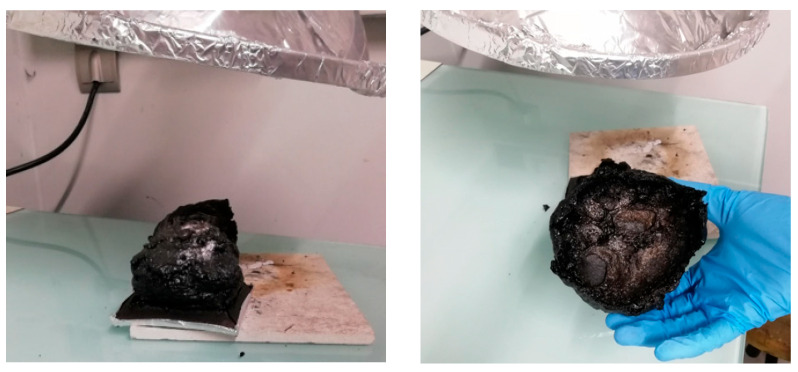
Sample showing signs of intumescence after testing in a cone calorimeter.

It should be noted that all tested formulations exhibited some degree of intumescence (Figure 5), likely due to the presence of -OH, -P, and -N groups. In fact, each of these groups plays a distinct role in the intumescence process. Specifically, -OH groups hydrate phosphorus compounds, promoting char formation. In addition, -P groups further dehydrate the matrix by removing the high-energy free radicals (-O, -OH, and -OH2) from the reaction, while -N groups act as foaming agents [33].

Once the individual performance of each bio-additive as a flame retardant and the synergism between them had been analysed, small coupons were developed by casting using the following formulations (Table 9):

The specimens prepared with the previous formulations were then characterised using the cone calorimeter.

The results in Table 10 indicate that the formulation containing 7 phr of phytic acid, 7 phr of tannic acid, and 1phr of chitosan (For 1.9) exhibited the most promising fire performance. This is due to the high concentration of phytic and tannic acids, which, as discussed in Section 1.2, promote the release of phosphorus during combustion and the formation of a carbonaceous layer, reducing flame propagation. Furthermore, phytic acid decomposes PO- and HPO radicals in the gas phase, capturing OH- and H- [34].

**Figure 6:**
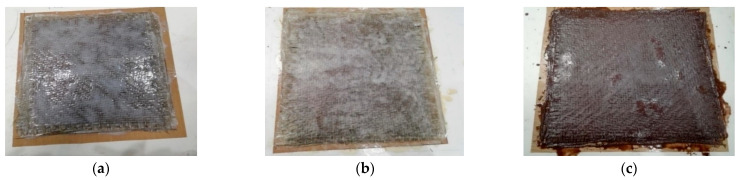
Hand-laminated composites using the developed formulations: (**a**) Reference; (**b**) For 1.9; (**c**) For 1.10.

After analysing the individual fire retardant behaviour of the bio-additives and their synergism, hand lay-up laminates were manufactured using 600 gr/m^2^ basalt fibre reinforcement with a resin–fibre ratio of 70% resin/30% fibre. The formulations used were the reference (For 1), and synergistic formulations 1.9 and 1.10 (Figure 6).

To ensure that the bio-based flame retardants did not inhibit the curing reaction, a DSC study was conducted on the formulations used for composite manufacturing.

**Figure 7:**
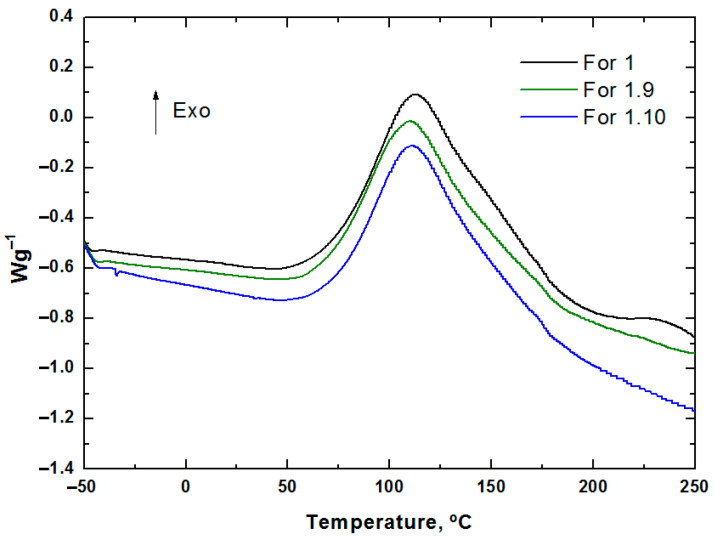
DSC results of the developed fire-retarded formulations.

The hand lay-up manufacturing technique employed in this study enables careful manual impregnation and air removal during lamination, ensuring good resin flow and compact laminate structures with minimal voids. This contributes to the achievement of a uniform distribution of the bio-based flame-retardant additives within the resin matrix while promoting an effective additive–resin interface, which is critical for maintaining mechanical integrity and consistent fire performance. Furthermore, the aforementioned DSC results (Figure 7) confirm that the addition of bio-based additives did not significantly influence either the maximum exothermic temperature or the curing enthalpy of the resin system, indicating that the additives are compatible with the resin and do not inhibit the curing reaction.

After curing and post-curing the developed composites, 100 mm × 100 mm coupons were taken for the cone calorimeter test (Table 11), yielding the following results.

Comparing the results obtained from the composites with the previously characterised castings, it can be seen that composites have a significantly higher MARHE value. Despite this increase, both the reference formulation (For 1) and the flame-retarded formulations met the MARHE requirement of <90 kW/m^2^. From the aforementioned results, it is worth noting that Formulation 1.9, which contains tannic acid, considerably improved the fire performance.

It is important to note that the MARHE values obtained for the fibre-reinforced composites (Table 11) are higher than those observed for the neat resin samples (Table 10). This behaviour is attributed to the presence of basalt fibre reinforcement, which alters the combustion behaviour, facilitating heat transfer within the specimen and limiting the efficiency of intumescence during combustion. Furthermore, the fibre reinforcement reduces the resin content per unit volume, decreasing the effective formation and expansion of the protective char layer that is more pronounced in neat resin samples. Despite this increase, all the developed composite formulations maintained MARHE values below the 90 kW/m^2^ limit required for railway sector applications, confirming their compliance with fire safety standards while demonstrating the viability of bio-based flame retardants in structural composite manufacturing.

#### 3.1.5. Mechanical Properties—Flexural Strength and Modulus

**Figure 8:**
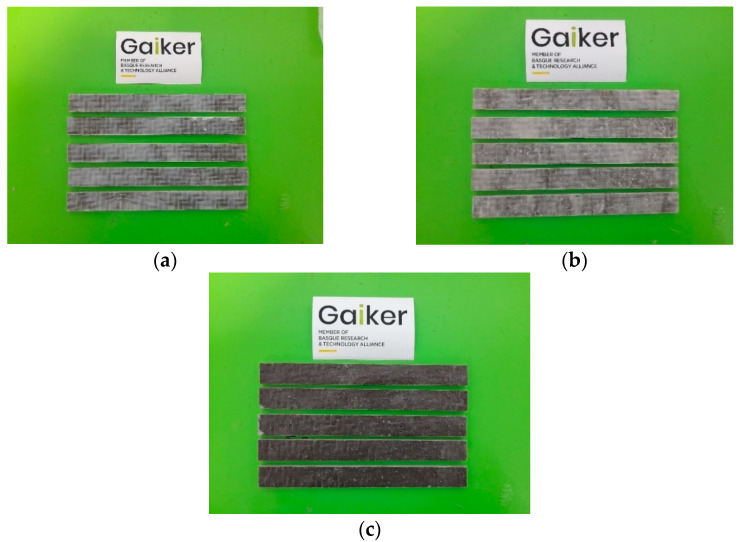
Coupons for mechanical properties made from basalt fibre and the developed fire-retarded formulations: (**a**) Reference; (**b**) For 1.9; (**c**) For 1.10.

Once the fire test of the laminated had been carried out, coupons of the remaining untested materials were taken for flexural testing (Figure 8).

The results in Table 12 indicate that all tested materials exhibited good flexural properties compared to conventional SMC materials used in the transport sector (flexural strength around 200 MPa). The laminates manufactured with the flame-retarded formulations showed a slight reduction in flexural strength, but without significant loss.

## 4. Conclusions

By way of conclusion, this study investigated the potential of bio-based additives as flame retardants in composite materials for the transport sector, with a focus on balancing fire resistance, mechanical properties, and processability.

Initial DSC characterisation of the resin systems revealed limited stability at room temperature, particularly for SMC applications. To mitigate premature reticulation, storing the optimised SMC formulations at sub-zero temperatures (approximately −18 °C) for future studies is recommended. Despite this limitation, DSC analysis indicated that the addition of bio-based flame retardants did not significantly alter the curing reaction or maximum exotherm temperature, suggesting compatibility with the thermoset matrix. The SR FireGreen 37 system, pre-selected for its inherent fire resistance, was prioritised for further evaluation.

On the other hand, cone calorimeter testing demonstrated that both the SR FireGreen 37 system and its bio-additivated variants exhibited promising fire performance, meeting critical requirements for the railway sector (MARHE < 90 kW/m^2^). Furthermore, synergistic combinations of flame retardants, particularly phytic acid, tannic acid, and chitosan (For 1.9), resulted in notable improvements in fire performance. These improvements are attributed to the combined effects of phosphorus release, char formation, and oxygen reduction, as evidenced by reduced MARHE and THR values.

Hand lay-up composites incorporating the optimised formulations exhibited proper flexural properties, with flexural strength values exceeding those of conventional SMC materials used in the transport sector. While the addition of flame retardants resulted in a slight reduction in flexural strength, the overall mechanical performance remained acceptable. The hand lay-up method also ensured good resin impregnation and laminate compactness, contributing to the uniform distribution of the bio-based additives within the matrix. DSC results further confirmed that the curing reaction proceeded correctly in the presence of the additives, supporting the compatibility between the resin and flame retardants without compromising the processability of the system. Notably, the developed composites achieved a high bio-based content (approximately 34% in the thermoset matrix) and enhanced fire resistance without compromising the curing reaction.

Overall, this work demonstrates that bio-based flame retardants offer a viable strategy for developing sustainable, low-toxicity composite materials with enhanced fire safety for the transport sector, aligning with environmental regulations and climate change mitigation strategies. Further research should focus on optimizing the long-term stability of the SMC formulations under refrigerated conditions for industrial scalability and exploring the performance of these materials under more realistic fire scenarios.

## Figures and Tables

**Table 1:** Parameters and maximum and minimum values for R1 of the EN 45545-2 standard [15].

Requirement Set (Relevant Product No.)	Test Method Reference	Parameter and Unit	Maximum or Minimum	HL1	HL2	HL3
R1 (IN1A; IN1B; IN1D; IN1E; IN4; IN5; IN6A; IN7; IN8; IN9B; IN11; IN12A; IN12B; IN14; F5	T02 ISO 5658-2: “Reaction to fire tests. Heat release, smoke production and mass loss rate”. Amendment 1. International Standard, Switzerland, 2019	CFE, kW/m^2^	Minimum	20	20	20
	T03.01 ISO 5660-1: 50 kW/m^2^	MARHE, kW/m^2^	Maximum	-	90	60
	T10.01 ISO 5659-2: 50 kW/m^2^	Ds (4), dimensionless	Maximum	600	300	150
	T10.02 ISO 5659-2: 50 kW/m^2^	VOF_4_, min	Maximum	1200	600	300
	T11.01 ISO 5659-2: 50 kW/m^2^	CIT_G_, dimensionless	Maximum	1.2	0.9	0.75

R: Requirement Set; HL: Hazard Level.

**Table 2:** Developed formulations (For) to study the reactivity of the epoxy systems, according to suppliers’ recommendations in parts per hundred resin (phr).

Bio-Based Epoxy System	%Bio-Content	For 1	For 2	For 3	For 4
SR FireGreen 37	25	100			
SD 8202		20			
SR GreenPoxy 33	26–28		100	100	
SD 4771			27		
SD 4770				27	
Polar Bear	20				100
R*Lab02					29

**Table 3:** Flame-retarded formulations (For) in phr.

Component	For 1	For 1.1	For 1.2	For 1.3	For 1.4	For 1.5
SR FireGreen 37	100	100	100	100	100	100
SD 8202	20	20	20	20	20	20
FR CROS484		15				
Chitosan			15			
Lignin				15		
Tannic Acid					15	
Phytic Acid						15

**Table 4:** DSC results showing the reaction progress of formulations stored over time.

Formulation	Time (h)	Max. Exothermic Temperature (°C)	∆H (J/g)	α (%)
For 1	0	115.0	391.3	0
	24	145.0	135.5	65.35
For 2	0	125.3	544.6	0
	24	137.6	118.9	78.16
For 3	0	128.3	515.9	0
	24	133.3	211.8	58.94
For 4	0	139.1	520.5	0
	24	137.0	371.4	28.65
	48	159.9	264.5	49.17

**Table 5:** Tg value determined by DSC.

Formulation	DSC Dynamic Scan	Tg (°C), DSC
For 1	−50 to 280 °C	112.61

DSC: Differential Scanning Calorimetry; Tg: Glass Transition Temperature.

**Table 6:** Viscosity of the developed formulations.

Formulation	Bio-Based Flame Retardant	Viscosity (mPa·s)
For 1	-	890 ± 10
For 1.1	FR CROS 484	1730 ± 100
For 1.2	Chitosan	16,900
For 1.3	Lignin	2420 ± 160
For 1.4	Tannic Acid	2530 ± 170
For 1.5	Phytic Acid	2500 ± 500

**Table 7:** Cone calorimeter results for For 4.

Formulation	Ignition Time (s)	Extinction Time (s)	MARHE (kW/m^2^)	THR 1200s (MJ/m^2^)	Qmax (kW/m^2^)	Mass Loss Rate (g/m^2^s)	TMLR (g/m^2^)
For 4	111	929	396.1	151	802	6.10	6635.9

**Table 8:** Cone calorimeter results for the developed formulations.

Formulation	Ignition Time (s)	Extinction Time (s)	MARHE (kW/m^2^)	THR 1200s (MJ/m^2^)	Qmax (kW/m^2^)	Mass Loss Rate (g/m^2^s)	TMLR 1200s (g/m^2^)	Maximum Intumescence (mm)
For 1	51	1170	43.8	36.8	95.1	2.27	2611.9	80
For 1.1	51	>1200	52.6	42.1	110.6	2.35	2715.2	80
For 1.2	45	>1200	34.7	35.8	99.8	2.54	2955.6	65
For 1.3	41	995	52.4	52.3	118.2	2.75	3220.5	90
For 1.4	29	>1200	28.9	32.4	76.8	2.63	3090.1	100
For 1.5	51	>1200	39.4	42.9	85.5	2.36	2677.9	75

MARHE: Maximum Average of Heat Emission; THR: Total Heat Release; Qmax: Maximum value of Heat Release; TMLR: Total Mass Loss Rate.

**Table 9:** New sustainable formulations designed to evaluate the synergistic effects of bio-based flame-retardant additives.

Formulation	Phytic Acid (phr)	Tannic Acid (phr)	Chitosan (phr)	Lignin (phr)
For 1.7	5	5	5	-
For 1.8	6	6	3	-
For 1.9	7	7	1	-
For 1.10	7	-	1	7

**Table 10:** Cone calorimetry results for formulations evaluating flame-retardant synergism.

Formulation	Ignition Time (s)	Extinction Time (s)	MARHE (kW/m^2^)	THR 1200s (MJ/m^2^)	Qmax (kW/m^2^)	Mass Loss Rate (g/m^2^s)	TMLR 1200s (g/m^2^)	Maximum Intumescence (mm)
For 1.7	45	1099	28	28.3	75.9	2.13	2419.6	90
For 1.8	41	771	35.9	37.8	77.2	2.20	2530.1	80
For 1.9	41	670	24.5	26.3	71.9	2.33	2758.7	85
For 1.10	47	778	35.1	32.1	81.7	2.41	2817	100

MARHE: Maximum Average of Heat Emission; THR: Total Heat Release; Qmax: Maximum value of Heat Release; TMLR: Total Mass Loss Rate.

**Table 11:** Fire behaviour of the most promising developed composites.

Formulation	Ignition Time (s)	Extinction Time (s)	MARHE (kW/m^2^)	THR 1200s (MJ/m^2^)	Qmax (kW/m^2^)	Mass Loss Rate (g/m^2^s)	TMLR 1200s (g/m^2^)	Maximum Intumescence (mm)
For 1	57	>1200	81.1	62.8	134.3	2.708	3102.9	90
For 1.9	49	971	72.9	46.4	118	2.406	2764.8	80
For 1.10	57	1197	83.1	46.5	149.4	2.354	2679.9	85

MARHE: Maximum Average of Heat Emission; THR: Total Heat Release; Qmax: Maximum value of Heat Release; TMLR: Total Mass Loss Rate.

**Table 12:** Mechanical properties (flexural strength and modulus) of the sustainable formulations developed.

Formulation	Flexural Modulus (MPa)	Flexural Strength (MPa)
For 1	9690 ± 692	302 ± 13
For 1.9	9260 ± 590	254 ± 16
For 1.10	8450 ± 576	245 ± 26

## Data Availability

The data reported in this study are available upon request from the corresponding author.

## Supplementary Materials

The following supporting information can be downloaded at: https://www.mdpi.com/article/10.3390/polym17152001/s1.
