# Supplementary material for: Effect of Bio-Based Flame Retardants in Sustainable Epoxy Systems for the Development of Composite Materials
Source: Polymers (Basel). 2025 Jul 22;17(15):2001. doi: 10.3390/polym17152001 (PMC12349320; doi:10.3390/polym17152001)
Supplement: Supplementary file 1 [file polymers-17-02001-s001.zip › polymers-3764524-supplementary.pdf]

SUPPLEMENTARY INFORMATION

In this document, additional information is contained from the article 'Effect of Bio-based Flame Retardants in Sustainable Epoxy Systems for the Development of Composite Materials'.

Table S1. Physicochemical properties of bio-based epoxy resins

Resin	Viscosity (25 °C, mPa·s)	Density (20 °C, g/ml)	Colour	%Bio-content
SR FireGreen 37	6000 ± 1200	1.27	white	24
GreenPoxy 33	1780	1.16	Clear	34-36
Polar Bear	11000-14000	1.16	Clear to light yellow	20

Table S2. Physicochemical properties of the hardeners

Hardener	Resin	Viscosity (25 °C, mPa·s)	Density (20 °C, g/ml)	Colour
SD 8202	SR FireGreen 37	25 ± 5	0.96	Light yellow
SD 4771 (ultra slow)<br>SD 4770 (mega slow)	GreenPoxy 33	9	0.94	Clear to yellow
R*Lab02	Polar Bear	5-15	-	Clear to yellow

Table S3. Chemical properties of bio-based additives

Property	Tannic Acid	Phytic Acid	Kraft Lignin	Chitosan
Natural origin	Vegetal	Rice	Vegetal	Crustacean shells
Formula	$C_{76}H_{52}O_{46}$	$C_6H_{18}O_{24}P_6$	-	-
Molecular weight (g/mol)	1701.20	660.04	-	50.000-190.000
Appearance and colour	Light beige powder	White powder	Brown powder	Faint Beige to Beige powder and/or chips
Zinc (%)	<0.005	-	-	-
Phosphorous content (%)	19-25	-	-	-

Table S4. Physicochemical properties of FR CROS484

Property	Value
Appearance	Fine, white powder
P2O5 content (%)	72
N-content (%)	14
Specific gravity (g/cm^3)	1.95
pH (10% in water)	5.5
Bulk Density (g/cm^3)	0.6
Solubility in water (g/100 cm^3)	0.8
Oil absorption (g oil/ 100 g)	27
Decomposition Temperature (°C)	300
Mean particle size d50 (µm)	18
Refuse at 45 µm (%)	1
Moisture (%)	0.05

Table S5. Physical properties of TBR-600® basalt reinforcement

Property	Value
Density (g/cm^3)	2.67
Range of work temperature (°C)	-250-650
Monofilament diameter (µm)	10-13
Humidity Content (% wt)	<0.5
Specific tensile strength (mN/tex)	>650
Grammage (g/m^2)	600
Watp (F/10 cm)	25
Weft (F/10 cm)	25
Thickness (mm)	0.6
